# Knowledge Architecture for Race and Ethnic Group Defining in Learning Health Systems

**DOI:** 10.1002/lrh2.70071

**Published:** 2026-02-26

**Authors:** Matthew F. Hudson, Virginia M. S. van Staden, Alicia M. Oostdyk, Julie C. Martin

**Affiliations:** ^1^ Prisma Health, Cancer Institute Greenville South Carolina USA

**Keywords:** culture, knowledge architecture, learning health system, quality, race, shared commitments

## Abstract

Previous work affirms access and health care challenges persist for multiple racial groups. Race and ethnicity are socially and politically constructed terms conceived to describe and categorize people hierarchically (race) and describe people from a similar national or regional background also sharing common national, cultural, historical, and social experiences (ethnicity). Definitions of race and ethnicity may vary across historical, political, and geographic contexts. This variation challenges health care organizations to reliably measure health care outcomes longitudinally within and between racial and ethnic groups. Further, suboptimal concordance between race and ethnicity self‐report vs. electronic health record suggests opportunities exist to improve race and ethnicity data solicitation and capture. Learning Health Systems (LHSs) may be particularly poised to address these challenges, as LHSs commit to equitable, transparent, and accountable engagement of individuals and families in care innovation. This discussion provides a rationale, model, and practical strategy to engage individuals and families in race data defining—an essential antecedent to health equity assessment and intervention development. The discussion encourages testing to ensure the proposed theory and steps beget comprehensive and precise race definitions. Enhanced precision in defining race may subsequently inform equity evaluation and interventions in LHSs.

## 1 Introduction

Earlier summaries [1] and more recent expositions [2, 3] reveal persisting health care access and quality challenges for select racial and ethnic groups. Race is a sociopolitical construct conceived to describe and categorize people hierarchically [4]; race is a dynamic social division that varies across historical, political, and geographic contexts [5, 6]. Ethnicity is a socially and politically constructed term used to describe people from a similar national or regional background and who also share common national, cultural, historical, and social experiences. Individuals often identify with or are assigned to an ethnic group based on a belief in shared ideas, values, behaviors, heritage, or language [6, 7]. Like race, ethnicity may also vary across historical, political, and geographic contexts. These “within‐individual” variations challenge health care organizations to reliably measure health care outcomes longitudinally within and between racial and ethnic groups.

Apart from “within individual” identification variation, earlier data collection instruments and methods potentially lacked precision and standardization. Consequently, the United States Office of Management and Budget refined requirements of federally‐funded agencies (e.g., select health care settings) to maintain, collect, and present data on race and ethnicity [8]. These revisions, though useful, may not optimize patient race and ethnic identification and data capture. Instruments (e.g., questionnaires) require implementation processes within systems. Thus, it is necessary to first consider infrastructure challenges and subsequently consider potential improvements that encourage robust, reliable, valid, and culturally‐relevant race and ethnic data collection and capture.

Health care infrastructure, meaning the resources required to execute health care, includes the built environment, equipment, access, information technology, systems and processes, sustainability initiatives and staff [9]. These resources should interact to optimize patient experience, effectiveness, efficiency, timeliness, safety, sustainability, and equity [9]. However, multiple examples suggest differential access to health care, and unwarranted treatment differences (see Yearby [10]). Structural racism‐a potential explanation for unwarranted treatment differences‐is the macrolevel systems, social forces, institutions, ideologies (e.g., assertions, theories) and processes that interact with one another to generate and reinforce inequities among groups [11]. To the extent a health care organization evidences structural racism, its existing data collection infrastructure may likewise exhibit compromised race and ethnicity data collection and capture ultimately portending flawed inferences [12, 13]. A flawed infrastructure may also explain Hasnian‐Wynia & Baker's [14] concern that patients may feel race and ethnicity queries undermine a perceived “caring relationship”. These concerns encourage race and ethnic data collection innovation. Specifically, care systems may need to recalibrate the health care infrastructure by creating processes that involve racial and ethnic groups in data collection processes. Diverse racial and ethnic involvement in data defining, collecting and capture may ultimately produce sounder race and ethnicity data.

Per earlier deliberations [15, 16] race and ethnicity data collection revisions may require a systems approach [17]. Further, Pettit et al. [18] encourage work examining how health care systems and patients collaborate to address how care systems record demographic information (e.g., race and ethnicity) in EHRs. We expand these considerations to further deliberate the type of system required to produce necessary changes to race and ethnicity data collection; we also more deeply consider how health care teams engage patients, families, and communities in data defining and recording.

## 2 A Learning Health Systems Approach to Race and Ethnicity Defining and Data Collection

The National Academies defines a learning health system (LHS) as a health system in which science, informatics, incentives, and culture are aligned for continuous improvement, innovation, and equity—with best practice and discovery seamlessly embedded in the delivery process, individuals and families active participants in all elements, and new knowledge generated as an integral by‐product of the delivery experience [19, 20, 21]. We contend the LHS definition encourages an infrastructure template particularly positioned to optimize race and ethnicity data defining, collection, and capture. First, the LHS, as defined by the National Academies, explicitly includes equity as a LHS outcome. Second, the definition also requires individuals and families to collaborate with care practitioners to advance all elements of the LHS, including equity. This LHS infrastructure template aligns with previous calls [18] for health care systems and patients to collaborate on race and ethnic data collection. Below, we explicitly define “equity.” Next, we scrutinize challenges defining and storing race and ethnicity data. Finally, we discuss how individuals (e.g., patients) and families are uniquely positioned to inform equity's definition and subsequent equity‐centric considerations. We ultimately propose a process model (a “how”) for defining and augmenting race and ethnic categories facilitating more comprehensive and accurate racial and ethnic group comparisons. We contend the individual and family engagement described in the process model may ultimately refine equity assessment. We also envision our process model guides LHS stakeholders toward actualizing individual and family partnerships in continuous improvement and innovation the LHS definition demands.

## 3 Equity Defined, Challenges to Defining Race and Ethnicity, and Opportunities to Improve Race and Ethnic Stratification

Health equity is the state in which everyone has a fair and just opportunity to attain their highest level of health [22]. Braveman et al. [23], health equity includes ethical and human rights principles that motivate people to eliminate health disparities‐the latter being avoidable differences in health or its key determinants (e.g., jobs offering fair compensation, quality education, housing, health care, and safe environments). The definition embeds both an assumption and supposition. The term “everyone” assumes multiple subpopulations (e.g., people who are members of subpopulations defined by racial or ethnic categories) exist within a principal population type (e.g., patient). This circumstance encourages individuals to examine inequity at the subpopulation level. Arcaya et al. [24] propose a group (or subpopulation) level inquiry is advantageous for targeting investments benefiting the less‐advantaged subpopulation. Arcaya et al. suggest group‐level inquiry (e.g., identifying subpopulations “A” and “B” and comparing average of continuous variable “Y” in “A” and “B”) can provide evidence for legislation and programming designed to eliminate social group differences. However, researchers observe, care access varies by subpopulations [10]. Therefore, group‐level equity inquiry challenges health care settings as care systems fail to admit all subgroup members; suboptimal access produces incomplete sets of subpopulations “A” or “B” and potentially admits only those with distinct characteristics or opportunities to gain access (producing a biased subpopulation of “A” or “B”). Given the possibility, hospital‐centric data underrepresents need in select populations, health service researchers may explore alternatives to traditional group‐level inquiry. Thus, Arcaya et al. alternatively explore the merits of overall health distribution in equity inquiry. This approach collapses all groups (or subpopulations) into one distribution. A health distribution approach predicates equity exploration on the outcome of interest (e.g., continuous variable “Y”), examining the outcome distribution, identifying suboptimal quartiles, identifying and comparing the characteristics of the suboptimal vs. optimal quartiles (e.g., percentage of subpopulations “A” and “B”). Arcaya et al. posit this approach potentially protects researchers from making incorrect assumptions about what social groupings (or subpopulations) matter in a particular circumstance. However, this approach may not clarify which (sub)population of interest fares better or worse and whether a preventable or (un)justified gap persists between the healthy and sick [24]. Further, suboptimal subpopulation coverage and bias vexes both overall health distribution analyses and group‐level analyses. Consequently, care facilities may require more comprehensive data to identify and stratify subpopulations. It is possible care facilities lack data on “key determinants”, such as a patient's employment compensation, education, or residence in a safe environment. Both conditions require more attention to how LHSs define the different populations they serve and engage stakeholders poised to additionally inform group definitions. Below, we will address challenges and opportunities to revise race and ethic data collection instruments and the infrastructure necessary to optimize race and ethnic data collection and capture.

The racial category people choose for themselves may change over time. Agadjanian [25] examined five panel surveys soliciting a nationally representative adult sample to self‐identify race several years apart. Agadjanian observed that 4% of individuals originally self‐identifying as White changed their race and about 20% of those originally self‐identifying in non‐white categories change their self‐identification across surveys. Non‐Hispanic Black people evidence an approximately 4% average race change rate. People originally coding Hispanics evidence an average change rate of 20%; People originally coding as Asians evidenced an average change rate of 12%. People reporting mixed race and “other” race evidence race change average rates of 52% and 73%, respectively. The variation Agadijanian observed exposes at least two potential threats to internal validity: History [26] refers to specific events (e.g., deportation threats) that occur between the first and second measurement potentially persuade self‐report change. Also, reflections on race may encourage an identification change (Maturation [26]). Apart from changes within the individual, measurement recalibrations [27] introduce a potential third internal validity threat when assessing race longitudinally (Instrumentation [26]). Measurement innovations may address some of these threats.

The United States Office of Management and Budget (OMB) provides federal agencies standard categories for collecting data on race and ethnicity [8, 28]. At present, OMB promotes the following categories: American Indian or Alaskan Native, Asian, Black or African American, Hispanic or Latino, Middle Eastern or North African, Native Hawaiian or Pacific Islander, and White. OMB solicitation also asks individuals to report multiple races and ethnicities. The Joint Commission (an international organization that accredits and certifies health care organizations and programs) [29] requires accredited hospitals to collect race and ethnicity data and encourages (but does not require) the use of OMB categories. The Joint Commission further encourages collecting ethnicity categories based on the population served [30]. Additionally, this accrediting and certifying body recently expanded these standards and guidelines to include ambulatory health, behavioral health, and critical access hospital services [30]. The Joint Commission cites resources encouraging race and ethnicity data collection as a necessary antecedent to advancing health equity [31, 32, 33]. Despite this guidance, Benjamin et al. [3] lament that United States health care systems do not uniformly collect, use, or report data on race and ethnicity, nor do care systems typically collect these data in a comprehensive or timely manner. Consequently, care facilities may encounter challenges assessing health care “fairness”, “justice” and patient‐centeredness within and between patient groups and care facilities.

Previous work also reveals a complex interplay of race, medical care, and geography. First, mere care availability is potentially related to the racial proportion of a given area. Gaskin et al. [34] observed the odds of being located in a primary care provider shortage area were 67% higher for United States zip codes with a majority Black persons population. However, zip codes with a majority Asian persons population and majority Latino persons population were less likely to be a named primary care provider shortage area. Second, suboptimal care in minority areas is not restricted to minorities. Baicker and colleagues [35] observed care quality Black patients received decreased as the population of people who are Black in an area increases; yet, they also observed this effect for people who are White. For example, the rate at which diabetics who are White received an annual eye exam also decreased as the percentage of people who are Black in an area increased. Consequently, care facilities aspiring to become “learning health systems” may need to expand inquiry beyond a patient's self‐assigned OMB category. “Learning” may require care systems to (at least) entertain (if not adopt) the premise that individuals and families may not self‐select a residential neighborhood nor its embedded risks and resources [36]. Informed by an ecosocial approach [37], LHS‐distinct equity explorations may augment traditional patient‐identified OMB category assessments and integrate residential segregation measures [38, 39]. These robust analytic approaches juxtapose “person” and “place” and may better elucidate the context driving patients' health and wellness experience. Stratifying racial groups, then, potentially requires two improvements: (1) patient group standardization and data collection reliability, (2) multidimensional considerations of care access and geographic characteristics. Considering race, neighborhood or service area's racial distribution, and resource availability may help learning health systems clarify race and health relationships. This more expansive consideration may also identify intervention targets LHSs may attack to ameliorate adverse relationships.

## 4 Challenges to Electronically Capturing/Storing Race and Ethnicity

Health services typically employ the electronic health record (EHR) to capture race and ethnicity data. However, EHR data may demonstrate inaccuracies. Earlier expositions [15] posit the lack of data collection standards contributed to misidentification of patient race. In geographies with more lax race data collection standards, administrative clerks' visual inspection or implementation of unstandardized patient race solicitation underestimated some groups, overestimated others, and over‐relied on the use of the “other” category compared to geographic areas utilizing a standardized race collection policy. This is important because even modest increases in data accuracy may enhance statistical power to observe true group difference obscured by misclassification bias. Modest increases in data accuracy may also provide statistical power to affirm the statistical significance of observed differences previously discarded owing to suboptimal sample size Klinger et al. [40] observed EHRs, when compared to self report as the gold standard, less reliably identified people who are Black (sensitivity 70.9%) than correctly identifying those people who are not Black (specificity 98.8%). Similarly, EHRs less reliability identified those people that were Hispanic (sensitivity 83.8%) than correctly identifying those people that were not Hispanic (specificity 99.8%). Thus, EHRs alone may be suboptimal means to identify patient populations. More recently, Johnson et al. [16] observed electronic health records (EHRs) have more incomplete and inaccurate data on patient race and ethnicity compared to disease registries. Johnson et al. observe databases have higher rates of misclassification or incomplete data for patients who are Hispanic compared to patients who are Whites and patients who are Black patients. People who are Asian, or Native American, or Pacific Islander are misclassified at the highest rates across most databases. Johnson et al. postulate EHRs, derived to facilitate healthcare transactions and workflows, lack the infrastructure required to have quality data for research. More recent single‐site studies demonstrate suboptimal concordance between EHRs and self‐report among Hispanic/Latino patients visiting a pediatric department [41] and an emergency department [18]. These findings justify the United States Office of Management and Budget's (OMB) 2024 call to revise definitions for minimum race and ethnicity reporting categories for all federal agencies and hospitals receiving federal funding [8]. Revisions include collecting race and ethnicity information using one combined question, adding “Middle Eastern or North African” as a new minimum category, and individuals can report more than one race. Revisions may standardize race and ethnicity reporting. However, the revision still “allow agencies flexibility to determine what additional data to collect to best meet program and stakeholder needs” (22186). How, then, may health care organizations take advantage of this measurement flexibility?

## 5 Acquiring, Managing, and Creating Knowledge About Race

We contend multiple independent but intertwined data are necessary to consider what a learning health system “knows” about race and its relevance to equitable care.

**FIGURE 1:**
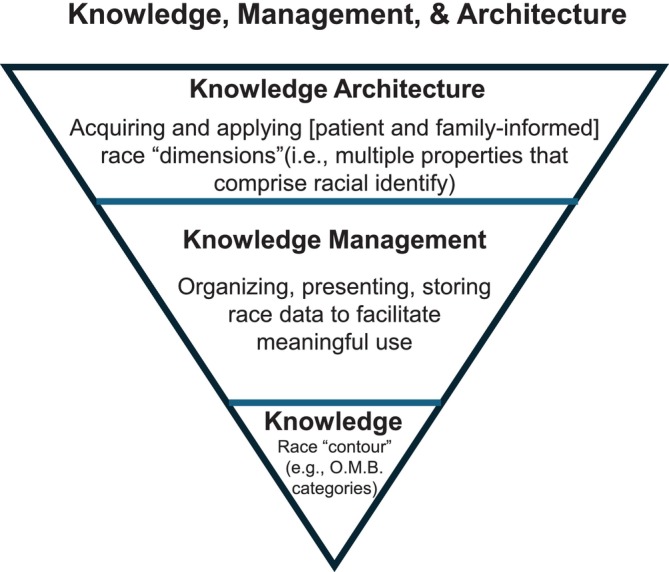
Knowledge, management, & architecture.

Figure 1 invites defining, managing, and planning for knowledge necessary to accurately define groups and subsequently discern health equity between these groups. Knowledge is the fact or condition of knowing something with familiarity gained through experience or association. We assert elemental knowledge is a “contour”‐an outline or superficial, yet socially meaningful attribute. In some instances, a contour may comprehensively or significantly represent a socially meaningful attribute (e.g., X‐ray of a bone fracture). In other instances, a contour may merely silhouette more complex attributes. For example, a patient‐reported OMB category outlines a socially meaningful attribute, but the attribute possesses multiple dimensions the contour (the OMB category) does not incorporate (e.g., community, environmental exposures, social ties, culture). In this latter example, knowledge users may erroneously presume the contour totally encompasses essential information. It is also possible knowledge users intentionally “settle” for a contour variable (e.g., “patient race as a proxy”) over a more precise composite variable. Precision aside, object familiarity gained through experience or association is a fundamental element we call knowledge. Care facilities must manage stakeholder and system knowledge to realize LHS aspirations.

Knowledge management involves knowledge production, presentation, storage, transfer and transformation. Knowledge architecture involve extraction of knowledge and knowledge management processes [42]. Knowledge management is akin to the LHS anchor feature, “Data Quality and Accessibility”. In our example, knowledge managers may “produce” data insofar as they solicit patients for information a patient may not otherwise provide (e.g., OMB racial classification). Knowledge managers may subsequently store, transfer, or present OMB data. Knowledge managers may transform OMB race data to facilitate interpretation (e.g., “transform” raw numbers into percentages). Although essential, knowledge management does not guarantee intentional data collection, team‐informed data, goal‐driven, or hypothesis‐driven data derivation or organization. Rather, we subsume knowledge and knowledge management under an overarching knowledge architecture that plans and coordinates knowledge application.

Knowledge Architecture is an organized framework instructing the acquisition and application of explicit knowledge for gaining success in competitive markets [42, 43]. Knowledge Architecture involves the extraction of knowledge and relevant knowledge managerial processes [42]. Knowledge Architecture incorporates the manner of creating knowledge, its application, and learning within an enterprise [42, 44].

**FIGURE 2:**
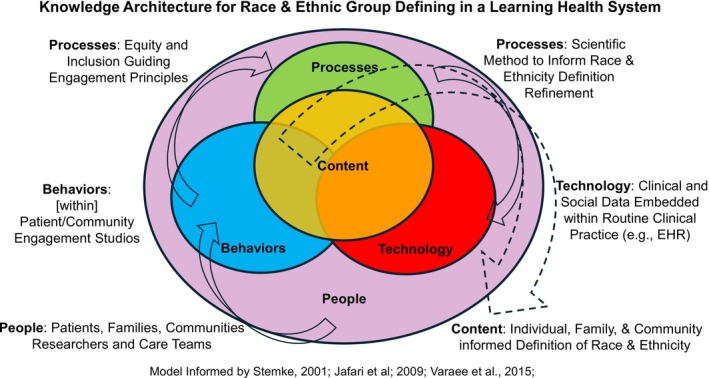
Knowledge architecture for race & ethnic group defining in a learning health system.

Per Figure 2, Knowledge Architecture illustrates how an LHS may intentionally design and organize the infrastructure to acquire information transitioning “contour” knowledge (e.g., OMB race category) to its fullest dimensions. Figure 2 proposes the following elements comprise the Knowledge Architecture: people (e.g., organization employees, individuals/patients, families/caregivers, and community members), processes (used by employees, patients, etc. to achieve organizational goals), behaviors (in settings where knowledge management must occur), technology (IT that facilitates knowledge detection, creation, and sharing within and outside the organization), and content (a shared knowledge database that can be electronically extracted) [42, 43, 45, 46]. Defining racial and ethnic (and other underserved) groups may require particular attention to the people and processes necessary to produce actionable content. We contend that patients and families are particularly essential partners LHS administrators should engage to optimally define race and ethnicity. We also propose a process for engaging patients and families in defining race.

## 6 The Importance of Partnering With Individuals and Families

Three reasons encourage health systems to partner with individuals and families to inform race and ethnic subgroup definitions. First, partnerships align with the LHS definition to engage individuals and families in all facets of improvement and innovation efforts. The engagement furthers a health care culture shift from siloed clinical practitioner responsibility and power to collective responsibility‐among public health, social services, businesses, community organizations, individuals and families‐for efforts to improve and innovate health care. Second, race is a social construct [47]. Given more genetic variation exists within self‐identified racial groups than between racial groups [48], medical technology (e.g., genes sequencing) and clinical expertise lack precision and relevance for distinguishing traditional race and ethnic categories. In contrast, individuals and families may provide insight into social and cultural attributes that more precisely refine racial and ethnic self‐defining and “othering”. Consequently, the National Academies and other scholars encourage researchers and health systems to partner with communities in race and ethnicity data derivation and collection [6, 49]. Third, select races and ethnicities are underrepresented in the health care workforce [50] and health care leadership, specifically. Meadows et al. [51], reports people who are Black and people who are Hispanic or Latino account for 3.5% and 5% of program directors, respectively. Similarly, people who are Black and people who are Hispanic and Latinos account for 4.2% and 3.7% of chairpersons, respectively. Two considerations underscore the relevance of the health care workforce racial distribution. First, the theory of the “relative heterogeneity effect” [52] posits members of an “in” group perceive their group as possessing diversity and conversely perceive an “out” group as more homogeneous. Thus, a given individual, regardless of group (e.g., race) affiliation may lack sensitivity to multiple race‐distinguishing characteristics for those outside their race (e.g., A person who is Hispanic is potentially more attuned to differences within populations who are Hispanic compared to populations that are Asian). Second, the “relative homogeneity effect” [52] posits members of a minority (in size/number) group will perceive less variability in their own group and more heterogeneity in non‐minority groups (e.g., people who are Black will perceive less diversity among people who are Black and more diversity among people who are White). The disproportionate racial representation in the health care workforce may encourage patient misclassification bias when workforce members (particularly majority members) perceive more within race variation in their own group compared to others. The small number of racial minorities in the health care workforce may over ascribe “sameness” to those within their own racial category. Both instances compromise the health care workforce's capacity to exclusively define and distinguish patient race. Thus, health care leaders may benefit from the depth and volume of social and cultural insights patients and community members can bring to inform race and ethnic definitions. Here, we do not propose that racial group categorization should be restricted to those who share the same race. Rather, we entertain the possibility a homogenous heath care workforce lacks representativeness beneficial for collectively considering the social, cultural, and geographic attributes comprising and distinguishing race and ethnicity. The three reasons potentially justify engaging individuals and families (and communities) in defining race and ethnicity. However, the reasons alone do not instruct care systems in how to engage individuals and families.

## 7 How Health Systems Can Involve Patients and Families

Care systems increasingly employ patient and community engagement studios [53, 54, 55, 56] to facilitate patient/researcher partnerships in the scientific method. Community engagement studios are structured groups that design processes to actively engage diverse stakeholders in team science [57]. The processes include research hypothesis development, study design, study implementation, and results dissemination. Figure 2 positions engagement studios not simply as an entity (or a resource), but a “way of doing research”. Hence, we model patient/community engagement studios as a behavior in the LHS knowledge architecture. Engagement studios concede collaboration does not occur automatically. Rather, optimal engagement is a product of gradual changes in comfort and perspective ultimately begetting transdisciplinary engagement. Per the LHS commitment to transparency [20], knowledge architecture processes should include targeted efforts to actualize fundamental engagement principles [58], notably: inclusion (creating a sense of belonging that recognizes and respects every party's perspective), equitable partnerships (co‐creation, co‐ownership, and shared decision making producing mutually appreciated outcomes), trust (considering the manner in which culture, individual and community experience impact assured reliance on partner(s)), trustworthiness (whether and how an individual demonstrates they are worthy of a given party's trust), and accountability (clarifying responsibilities, providing reliable feedback [20], and sharing a commitment to overcoming barriers to progress [58]). We believe these engagement principles are especially important in race and ethnicity deliberations.

Upon establishing the noted engagement principles, the Knowledge Architecture may encourage patients, researchers, clinicians, and administrators to execute the following explicit steps, rooted in the scientific method, to innovate race and ethnic categorization and use for equity assessment: *Observation*: Identify patient care and care‐independent circumstances motivating the health system to collect race and ethnicity data. Ascertain whether current data categories concord with OMB policy. Identify collection procedure (e.g., self‐report, observation). Review the literature to consider identified challenges in existing race and ethnicity data [59]. The literature review may collectively inform the group of persisting challenges, “level set”, and provide a benchmark for understanding existing data collection methods and interpreting their product. *Hypothesis Development*: patient/family/researcher teams may reference conceptual models to discern whether the collective interest lies in questions of race [60], racism [61], or structural racism [62]. Such deliberation may help refine knowledge informing racial categories and whether to isolate other variables race may loosely proxy (e.g., income, neighborhood). *Method*: Research partners may initially define the patient or community population via data frequently captured in the electronic health record. Alternatively, the nature and purpose of the deliberation may encourage stakeholders (i.e., knowledge architects) to refine or innovate data collection to ensure the system collects salient information in the course of routine clinical practice (an LHS attribute). Knowledge architects may also describe the instrument and tools they use to subsequently solicit information germane the architects' refined race definition. Knowledge architects may then use responses to create patient profiles predicated upon the newly acquired information. *Analysis*: The knowledge architects may first consider any overlap between existing race categories (e.g., OMB categories) and new research team‐created profiles. The knowledge architects may compare the novel and preexisting race measures' sensitivity and specificity to identifying outcomes of interest (e.g., disease, prognosis, treatment preference). *Results Dissemination*: Individual and family research partners are particularly positioned to share the results of, for example, a patient‐centered classification of race vs. an OMB‐endorsed classification method. Individuals/patients and family research partners can extend the findings beyond the clinical confines to affirm any race definition innovations with the communities that individuals and families represent. *Hypothesis Refinement*: Feedback the knowledge architects receive, particularly from communities, can beget measure refinement or procedural refinement. Measure refinement may restructure questions knowledge architects pose to obtain information. Procedural refinement may expand or restrict when, where, and how knowledge architects solicit information. Hypothesis refinement may also encourage technological refinement to the electronic health record. Hypothesis refinement may also direct technological innovation of artificial intelligence toward health disparities address [63].

## 8 Discussion

The definition of race and ethnic groups will continue evolving. We attribute this continual evolution to individual, social, and political shifts (sometimes intended to elevate one group over another). Motivations notwithstanding, the knowledge architecture potentially requires an “adaptive capacity” [64] to account for and adjust to evolving and expanding definitions in real time. This evolution potentially includes increased sensitivity to intersectionality [65]‐where multiple attributes of identity (e.g., race and sex) overlap and interact potentially creating distinct or compound forms of discrimination. Thus, health care systems (and their knowledge architecture) myopically focused on care equity by race and/or ethnicity potentially compromise opportunities to more comprehensively consider how those possessing membership in multiple historically marginalized groups experience a unique or compound discrimination effect. For example, women who are black report unfair treatment, and practice vigilant behaviors (e.g., prepare for possible insults), more than black men and white women [66]. These findings potentially expose multiple dimensions of discrimination underscoring the need for a diverse LHS knowledge architecture. This is particularly important if one adopts the perspective that intersectionality is not capricious. Rather, the knowledge architecture positions LHSs to disassemble intentional, *structural* (e.g., how parties organize institutions), *disciplinary* (e.g., how parties operate institutions), *hegemonic* (e.g., social and cultural influence empowered parties impose) and *interpersonal* (e.g., the routinized practice of how parties treat each other) elements potentially comprising a “matrix of domination” [67] intentionally oppressing select groups.

Adapting to race and ethnicity definition challenges in real time requires the knowledge architecture be willing to *reframe* the way it expresses, considers, or performs race and ethnicity defining and inquiry, *align* efforts to convergence, shared meaning, and practices to form a shared outcome (i.e., negotiate different interests to arrive at decisions); race and ethnicity‐based deliberations will also require *coping* with both internal and external demands so as to produce race and ethnicity definition *innovation* [64]. Executing these activities requires the knowledge architecture be capable of integrating and combining different knowledge sources, coordinating resources, communicating frequently (preferably “face to face”) and trusting individual members and the collective knowledge architecture [64]. LHS leaders invested in facilitating the knowledge architecture's adaptative capacity should build competence (e.g., facilitate training and monitor/evaluate learned training and skills), balance workload and staff needs (e.g., knowledge architecture activities vis a vis clinical responsibilities), demonstrate engagement and investment in activities (e.g., build an esprit de corps begetting knowledge architecture empowerment and personally observe activities to understand opportunities and challenges), and situational understanding of work and practice needs (e.g., create surroundings where knowledge architecture can execute work seamlessly and efficiently) [68].

Our discussion magnifies the LHS definition's admonition to involve individuals and families in all facets of improvement and innovation. Health systems struggling with either a value‐added “entry point” or engagement strategy for individuals and families may reference the discussion above to optimize individual and family engagement in fundamental equity considerations. However, knowledge architects (e.g., individuals/families, health practitioners, administrators, embedded researchers) may be ill‐equipped to transparently consider why and how we examine race [69]. The topic requires profound introspection and vulnerability engagement principles encourage. Each health system should discern their amenability to the National Academies shared commitments [20], specifically transparency and accountability. Transparency, per the National Academies, requires full clarity and sharing in activities, processes, results, and reports. Here, transparency may particularly require a commitment to reveal discomfort that frequently accompanies discussions about race and ethnicity. Accountability, per the National Academies, refers to identifying and addressing clear responsibilities, using measures that matter, and providing reliable feedback. Here, accountability may particularly demand partners constructively consider how their values fundamentally (and potentially subconsciously) inform problem identification (e.g., illness) and investigation regarding potential correlates (e.g., race) and solutions. Health systems committed to the spirit and intent of “learning” about how to best define race and ethnicity may experience great stress. This stress may portend tepid engagement, withdrawal from team processes, and ultimately fracture teams. However, our discussion, the National Academies LHS definition, and its Shared Commitments provide a framework to endure the stress. Our proposed model and approach may produce greater precision in defining race, and engaging individuals and families in that process.

## 9 Summary

To best address variation in race and ethnicity data reporting and capture, we proposed LHSs employ the theoretical model and practical steps we described to refine the definition of racial and ethnic groups. We argue these steps necessarily precede health equity considerations. We argued that including individuals and families in race and ethnicity‐defining efforts may ultimately produce more tenable equity assessments. We concede integrating individuals and families (and communities) into the LHS Knowledge Architecture may particularly require transparency and accountability—two shared commitments of the LHS. Undertaking these proposed strategies will produce data, technology, and analytic refinement ultimately directing intervention development and implementation for care equity. We welcome empirical testing of our assumptions to ensure the proposed strategy truly realizes the LHS aspiration.

## Funding

The authors have nothing to report.

## Disclosure

The Lead/Corresponding author is a member of the Editorial Board. Neither he nor the co‐authors will be involved in this manuscript's editorial review or inform publication decisions.

## Conflicts of Interest

The authors declare no conflicts of interest.

## Data Availability

Data sharing not applicable to this article as no datasets were generated or analysed during the current study.
